# Myocardial fatty acid uptake through CD36 is indispensable for sufficient bioenergetic metabolism to prevent progression of pressure overload-induced heart failure

**DOI:** 10.1038/s41598-018-30616-1

**Published:** 2018-08-13

**Authors:** Yogi Umbarawan, Mas Rizky A. A. Syamsunarno, Norimichi Koitabashi, Hideru Obinata, Aiko Yamaguchi, Hirofumi Hanaoka, Takako Hishiki, Noriyo Hayakawa, Motoaki Sano, Hiroaki Sunaga, Hiroki Matsui, Yoshito Tsushima, Makoto Suematsu, Masahiko Kurabayashi, Tatsuya Iso

**Affiliations:** 1 Department of Cardiovascular Medicine, Gunma University Graduate School of Medicine, Maebashi, Gunma 371-8511 Japan; 2 Department of Bioimaging Information Analysis, Gunma University Graduate School of Medicine, Maebashi, Gunma 371-8511 Japan; 3 Department of Diagnostic Radiology and Nuclear Medicine, Gunma University Graduate School of Medicine, 3-39-22 Showa-machi, Maebashi, Gunma 371-8511 Japan; 4 Department of Laboratory Sciences, Gunma University Graduate School of Health Sciences, 3-39-22 Showa-machi, Maebashi, Gunma 371-8511 Japan; 5 Research Program for Diagnostic and Molecular Imaging, Division of Integrated Oncology Research, Gunma University Initiative for Advanced Research (GIAR), Maebashi, Gunma 371-8511 Japan; 6 Gunma University Initiative for Advanced Research (GIAR), 3-39-22 Showa-machi, Maebashi, Gunma 371-8511 Japan; 7 Department of Biochemistry, Keio University School of Medicine, Shinjuku-ku, Tokyo 160-8582 Japan; 8 Clinical and Translational Research Center, Keio University School of Medicine, Shinjuku-ku, Tokyo 160-8582 Japan; 9 Department of Cardiology, Keio University School of Medicine, 35 Shinano-machi, Shinjuku-ku, Tokyo 160-8582 Japan; 10 Department of Biochemistry and Molecular Biology, Universitas Padjadjaran, Jl. Raya Bandung Sumedang KM 21, Jatinangor, West Java, 45363 Indonesia; 11 Department of Internal Medicine, Faculty of Medicine Universitas Indonesia, Jl. Salemba Raya no. 6, Jakarta, 10430 Indonesia

## Abstract

The energy metabolism of the failing heart is characterized by reduced fatty acid (FA) oxidation and an increase in glucose utilization. However, little is known about how energy metabolism-function relationship is relevant to pathophysiology of heart failure. Recent study showed that the genetic deletion of CD36 (CD36KO), which causes reduction in FA use with an increased reliance on glucose, accelerates the progression from compensated hypertrophy to heart failure. Here, we show the mechanisms by which CD36 deletion accelerates heart failure in response to pressure overload. CD36KO mice exhibited contractile dysfunction and death from heart failure with enhanced cardiac hypertrophy and interstitial fibrosis when they were subjected to transverse aortic constriction (TAC). The pool size in the TCA cycle and levels of high-energy phosphate were significantly reduced in CD36KO-TAC hearts despite an increase in glycolytic flux. De novo synthesis of non-essential amino acids was facilitated in CD36KO-TAC hearts, which could cause a further decline of the pool size. The ingestion of a diet enriched in medium-chain FA improved cardiac dysfunction in CD36KO-TAC hearts. These findings suggest that myocardial FA uptake through CD36 is indispensable for sufficient ATP production and for preventing an increased glycolytic flux-mediated structural remodeling during pressure overload-induced hypertrophy.

## Introduction

The heart requires constant synthesis of enormous amounts of ATP to sustain cellular processes such as excitation-contraction coupling and ion homeostasis within the cardiac myocytes. In adult cardiac myocytes, most ATP production (>95%) occurs through the process of mitochondrial oxidative phosphorylation, approximately 70–90% of which is derived from fatty acid (FA) oxidation and the remaining from glucose, lactate, amino acid, and ketone oxidation^1–4^.

The failing heart is associated with a decline in FA oxidation while reliance on glucose is increased^1,3,4^. This shift of substrate preference from FA to glucose in failing heart has been considered adaptive to diminish oxygen consumption. Therefore, it has been proposed that reducing cardiac FA utilization and/or an increase in glucose use may be possible therapeutic strategies for heart failure. Various metabolic interventions and animal models have been employed to address this possibility^1,3,4^. However, the evidence thus far suggests that long term effects of such modification in metabolism and cardiac function are still controversial.

Tricarboxylic acid (TCA) cycle is a metabolic hub to link a number of catabolic and anabolic pathways^5,6^. Because total amount of intermediates in the TCA cycle (pool size) are crucial for the normal functioning^6^, influx of metabolites into the TCA cycle are exquisitely regulated by various feedback systems including glucose-fatty acid cycle (also known as Randle cycle)^7^. In addition to ATP synthesis, components of the TCA cycle form essential links with the anabolic pathways for gluconeogenesis, lipogenesis and synthesis of non-essential amino acids, that are facilitated depending on cell types, energy status and pathophysiological situations^4,8,9^.

CD36, also known as fatty acid translocase (FAT), is a single chain 88-kDa glycoprotein that has wide biological functions in various kinds of cells, such as adipocyte, macrophage, endothelium and skeletal and cardiac muscle^10^. In the heart, CD36 has a major role in FA uptake^11,12^. Under resting conditions, the heart in CD36 knockout (CD36KO) mouse displays reduced rates of FA transport and oxidation with marked enhancement of glucose use. It was recently reported that cardiomyocyte-specific deletion of CD36 accelerates cardiac contractile dysfunction in response to pressure overload. Feeding these CD36KO mice a diet enriched in medium-chain fat, which bypasses CD36 for entry into the cardiomyocyte, is able to protect these mice from developing heart failure^13^. These findings suggest that the detrimental effect of CD36 ablation may be caused by energy insufficiency, which reminded us of well-known energy starvation theory as pathogenesis of heart failure^14^. However, cardiac metabolism (catabolism and anabolism) and energetic status under pressure overload *in vivo* remains unanswered.

In the present study, through a series of metabolic profiling and isotopomer analysis, we demonstrate that CD36KO mouse heart subjected to pressure overload displays accelerated cardiac dysfunction accompanying a diminished pool size of the TCA cycle despite a robust increase in glycolytic flux into the TCA cycle. Together with the findings that the increased glucose consumption is used as a carbon source for biomass synthesis including amino acids, our data provide the mechanistic insight into a link between myocardial ATP decline and pathological hypertrophy during pressure overload.

## Results

### Severe left ventricular systolic dysfunction in CD36KO mice in response to TAC

**Figure 1:**
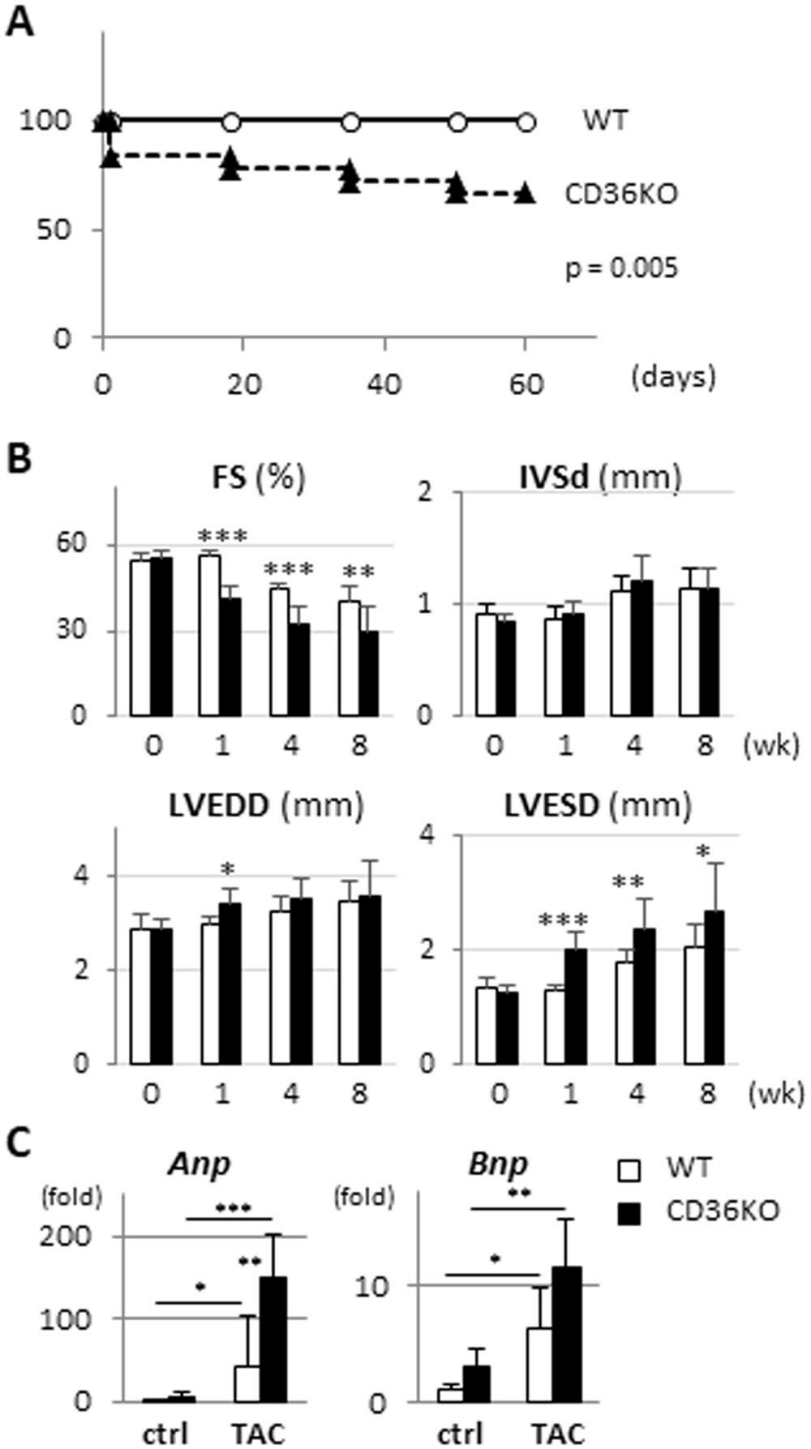
Cardiac contractile dysfunction was more deteriorated in CD36KO-TAC mice from early time point with reduced survival rate. (**A**) Survival curves of WT and CD36KO mice after TAC. Survival rate was significantly reduced in CD36KO-TAC mice (n = 18–19). p = 0.005. (**B**) Cardiac function was estimated by echocardiography before and 1, 4, and 8 weeks after TAC. Cardiac contraction was reduced in CD36KO mice with enlarged LV systolic diameter after TAC (n = 10–12). FS, fractional shortening; IVSd, thickness of interventricular septum in diastole; LVEDD, left ventricular end-diastolic diameter; LVESD, left ventricular end-systolic diameter. (**C**) Expression of cardiac stress marker genes, *Anp* and *Bnp*. *Anp*, atrial natriuretic peptide; *Bnp*, brain natriuretic peptide (n = 6). ^*^p < 0.05, ^**^p < 0.01, ^***^p < 0.001.

To study whether the substrate shift from FA to glucose influences the cardiac function in the context of increased workload, mice were subjected to transverse aortic constriction (TAC). Six out of 18 CD36KO-TAC mice died by 8 weeks after TAC, but no mice died in WT-TAC. Survival rate was significantly reduced in CD36KO-TAC mice (Fig. 1A). Echocardiographic analysis revealed that while no functional difference between CD36KO and WT mice was observed at baseline, diminished % fractional shortening and increased LV diastolic and systolic chamber diameter was more prominent in CD36KO-TAC mice all measured time points after 1 week (Fig. 1B and Table S1). Consistent with these results, an inducible expression of cardiac stress markers, *Anp* and *Bnp*, was greater in CD36KO mice (Fig. 1C). These findings suggest that contractile failure in CD36KO-TAC mice is more accelerated compared to that in WT-TAC from early time point after TAC.

### Marked structural remodeling in CD36KO mice in response to TAC

**Figure 2:**
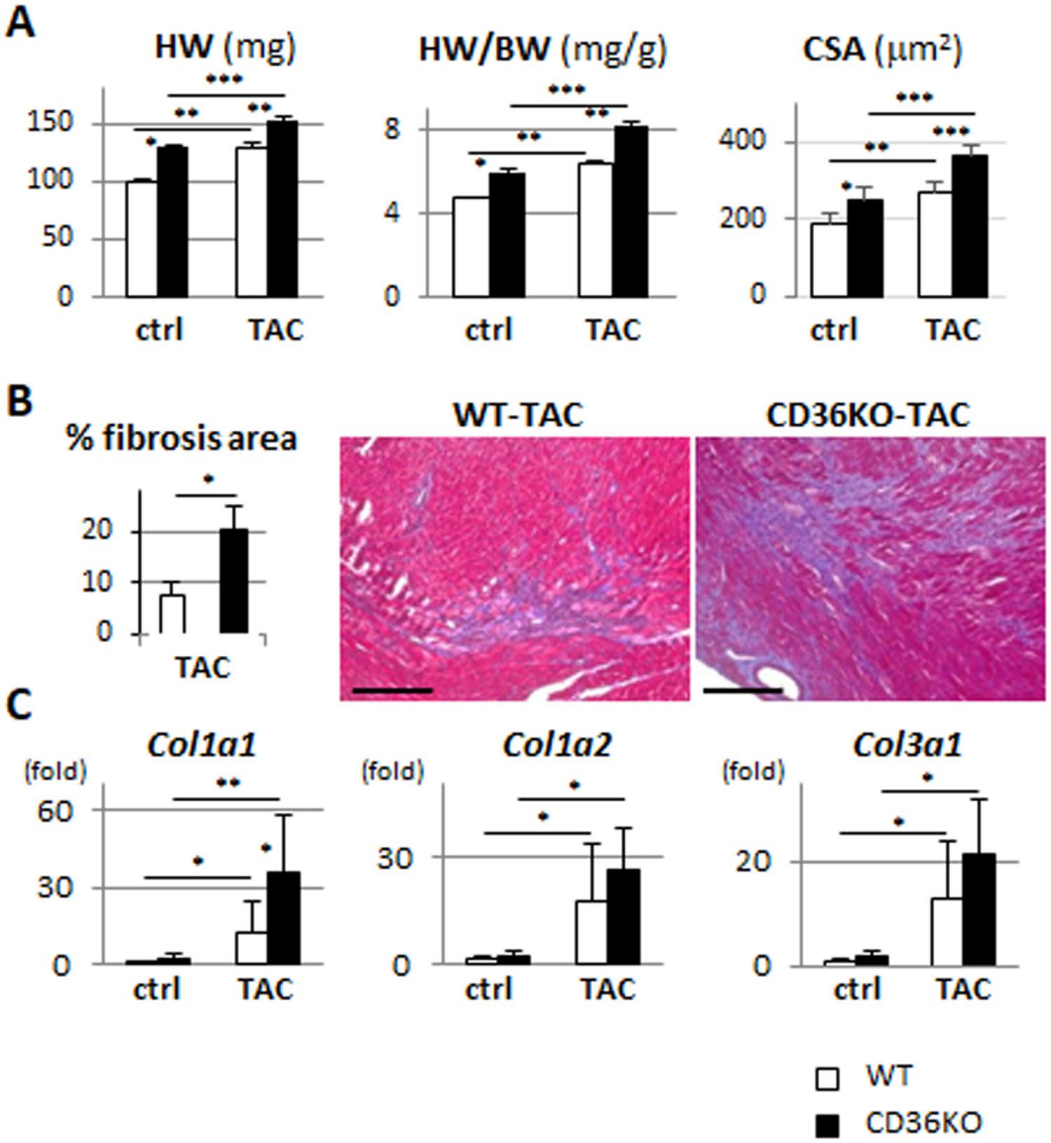
Cardiac Hypertrophy and fibrosis was enhanced in CD36KO-TAC hearts. (**A**) Heart weight/body weight ratio (HW/BW) and cross sectional area were increased in CD36KO-TAC hearts (n = 6). (**B**) Fibrosis area estimated by Masson Trichrome stain was increased in CD36KO-TAC hearts (n = 6). Scale bar = 200 μm. (**C**) Expression of mRNA for collagen fibers in hearts. *Col1a*, collagen 1a. ^*^p < 0.05, ^**^p < 0.01, ^***^p < 0.001.

We studied structural remodeling in response to pressure overload 1 week after TAC when significant difference of cardiac function between WT-TAC and CD36KO-TAC mice was observed. Interestingly, cardiac hypertrophic response in CD36KO mice was more prominent as indicated by a significant increase in heart weight to body weight ratio and cell surface area (Figs 2A and S1). Fibrosis area was also significantly increased in CD36-TAC hearts compared to WT-TAC (Fig. 2B), which was accompanied by an increase in expression of collagen fiber genes, *Col1a1*, -*1a2* and -*1a3* (Fig. 2C). These data showed that CD36KO mice have more prominent hypertrophic and fibrotic remodeling in response to pressure overload.

### CD36KO hearts heavily rely on glucose irrespective of pressure overload

**Figure 3:**
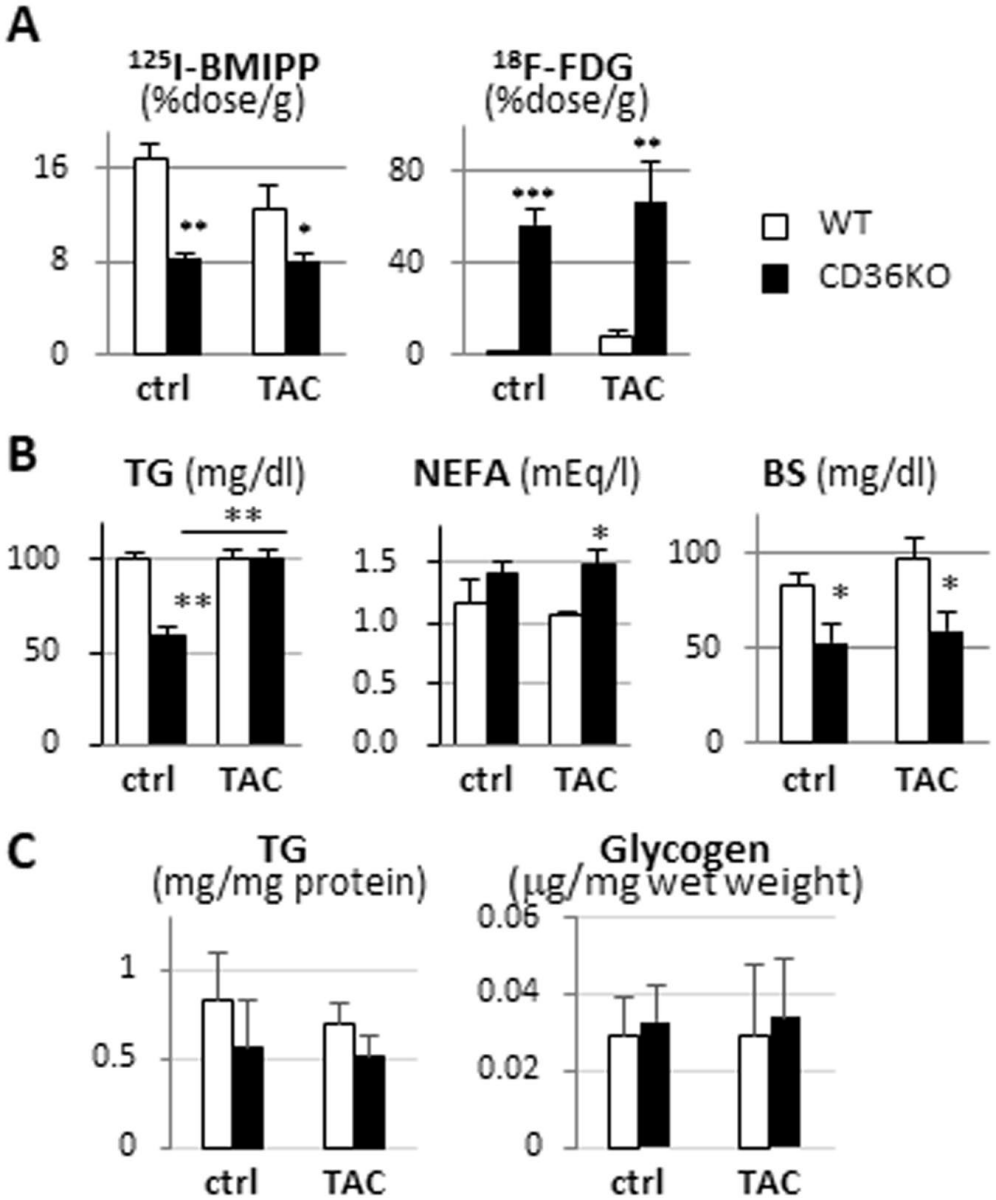
Uptake of FA and glucose by hearts, circulating levels and cardiac accumulation of lipids and glucose in the absence or presence of TAC. Samples were collected after a 12 h fast. n = 5–7. (**A**) Uptake of ^125^I-BMIPP, FA tracer, and ^18^F-FDG, glucose tracer, by hearts. (**B**) Serum levels of TG, NEFA and glucose. (**C**) TG and Glycogen contents in hearts. TG, triacylglycerol; NEFA, non-esterified fatty acids. ^*^p < 0.05, ^**^p < 0.01, ^***^p < 0.001.

We next examined the effects of TAC on uptake of ^125^I-BMIPP, FA tracer, and ^18^F-FDG, glucose tracer. ^125^I-BMIPP uptake was lower in CD36KO hearts by 50% compared to WT at baseline (Fig. 3A). Following TAC surgery, ^125^I-BMIPP uptake was reduced by 25% in WT hearts while no further reduction was observed in CD36KO hearts. Serum TG levels were comparable between WT-TAC and CD36KO-TAC mice while serum NEFA levels were higher in CD36-TAC mice (Fig. 3B). TG levels, a storage form of FA, tended to be lower in CD36KO hearts before and after TAC, although the differences were not significant (Fig. 3C). ^18^F-FDG uptake was 35-fold higher in CD36KO hearts compared to WT at baseline to compensate for reduced FA uptake (Fig. 3A). ^18^F-FDG uptake tended to be increased in both groups after TAC (Fig. 3A). Consistent with a robust increase in ^18^F-FDG uptake in CD36KO hearts, blood glucose levels were lower in CD36KO mice than those in WT (Fig. 3B). Glycogen levels, a storage form of glucose, were not different between groups with or without TAC (Fig. 3C). These findings strongly suggest that CD36KO hearts heavily rely on glucose before and after TAC.

### Expression of genes involved in FA use was similarly repressed by TAC in WT and CD36KO mice

**Figure 4:**
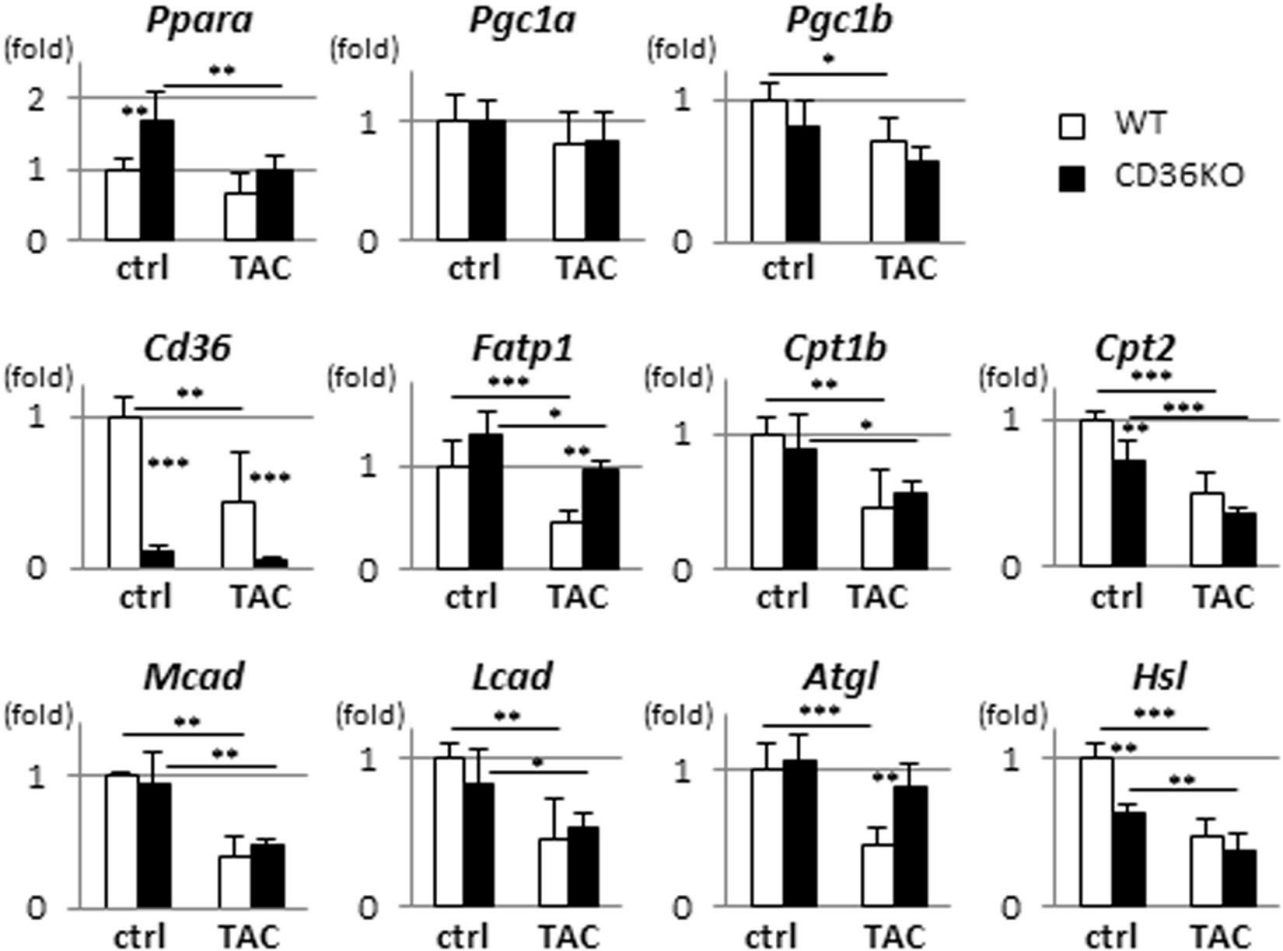
Expression of genes associated with FA metabolism in hearts. Hearts were isolated after a 12 h fast for quantitative polymerase chain reaction (n = 6). *Ppara*, peroxisome proliferator activated receptor α; *Pgc1a/b*, PPARG coactivator 1 alpha/beta; *Fatp1*, fatty acid transport protein 1; *Cpt1b/2*, carnitine palmitoyltransferase 1B/2; *Mcad*, medium-chain acyl-CoA dehydrogenase; *Lcad*, long-chain acyl-CoA dehydrogenase; *Atgl*, adipose triglyceride lipase; *Hsl*, hormone sensitive lipase. ^*^p < 0.05, ^**^p < 0.01, ^***^p < 0.001.

We next examined expression profile of genes involved in FA utilization by quantitative PCR. Expression of mRNA for peroxisome proliferator-activated receptor α (*Ppara*) and its co-activators, PPARγ-coactivator-1 (*Pgc1a* and *Pgc1b*), three of which are central regulators of FA uptake and oxidation, tended to be decreased in WT-TAC and CD36KO-TAC to the same degree (Fig. 4). Consistent with these findings, the expression of the genes regulated by *Ppara/Pgc1* complex, such as *Cpt1b*, *Cpt2*, *Mcad*, and *Lcad*, were decreased by TAC in both mice. The expression of the gene, *Hsl*, for lipolytic enzyme was decreased in both mice while the expression of another lipolytic enzyme, *Atgl*, was not suppressed in CD36KO-TAC. Expression of *Cd36* was negligible in CD36KO mice while its expression was significantly suppressed in WT-TAC mice compared to that at baseline. Thus, there was no further suppression of gene expression involved in FA use in CD36KO-TAC hearts compared to WT-TAC except for CD36.

### Accelerated glycolysis in CD36KO hearts at baseline and after TAC

**Figure 5:**
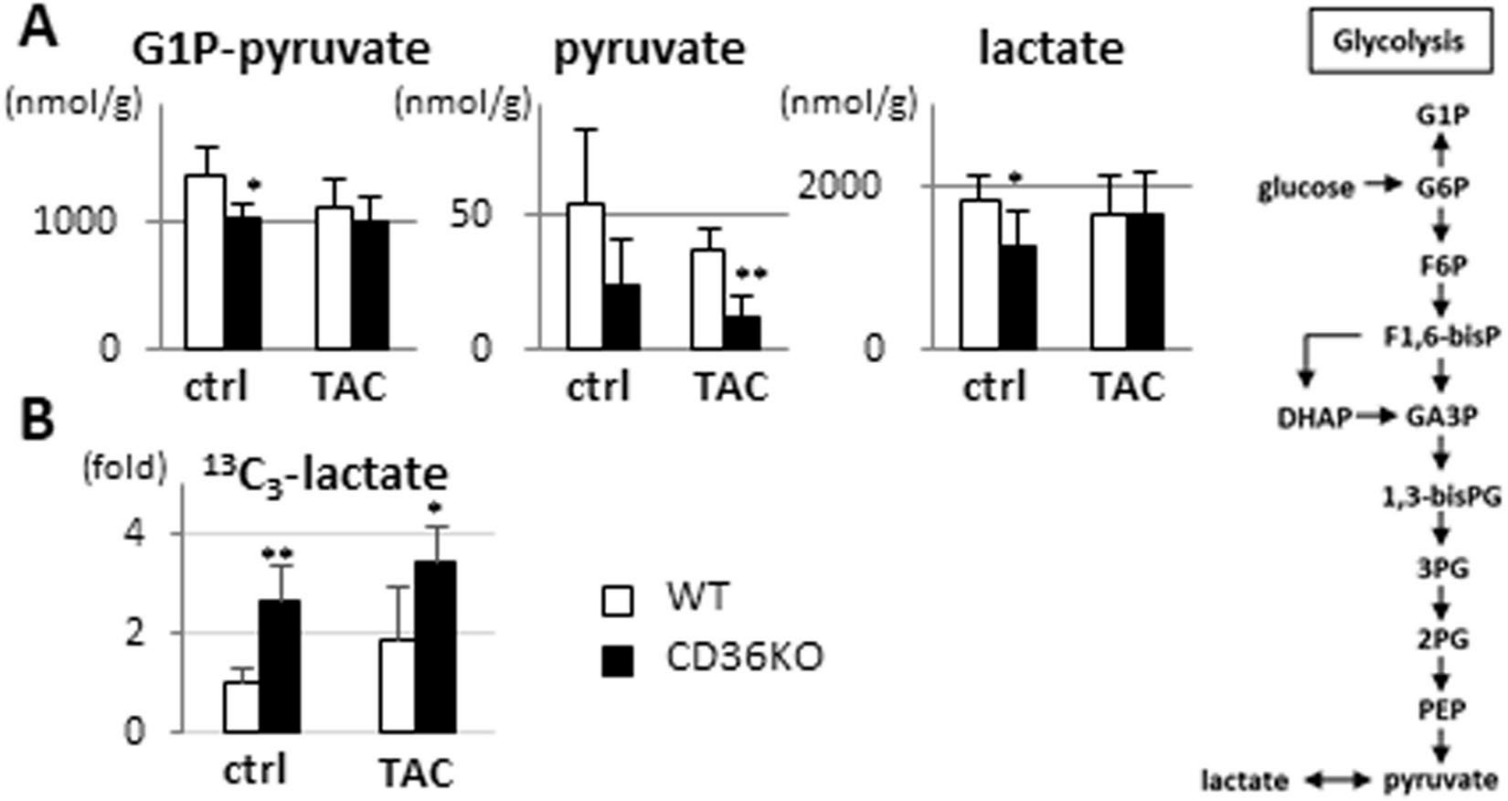
Metabolic profiling and its flux in glycolysis. (**A**) Metabolic profiling in glycolysis pathway. G1P, glucose-1-phosphate. G1P-pyruvate, total metabolites from G1P to pyruvate in glycolysis (n = 5–7). (**B**) Tracer study with ^13^C_6_-glucose. After a 12 h fast, hearts were isolated 10 min after intraperitoneal injection of ^13^C_6_-glucose (n = 5–7). ^*^p < 0.05, ^**^p < 0.01.

**Figure 6:**
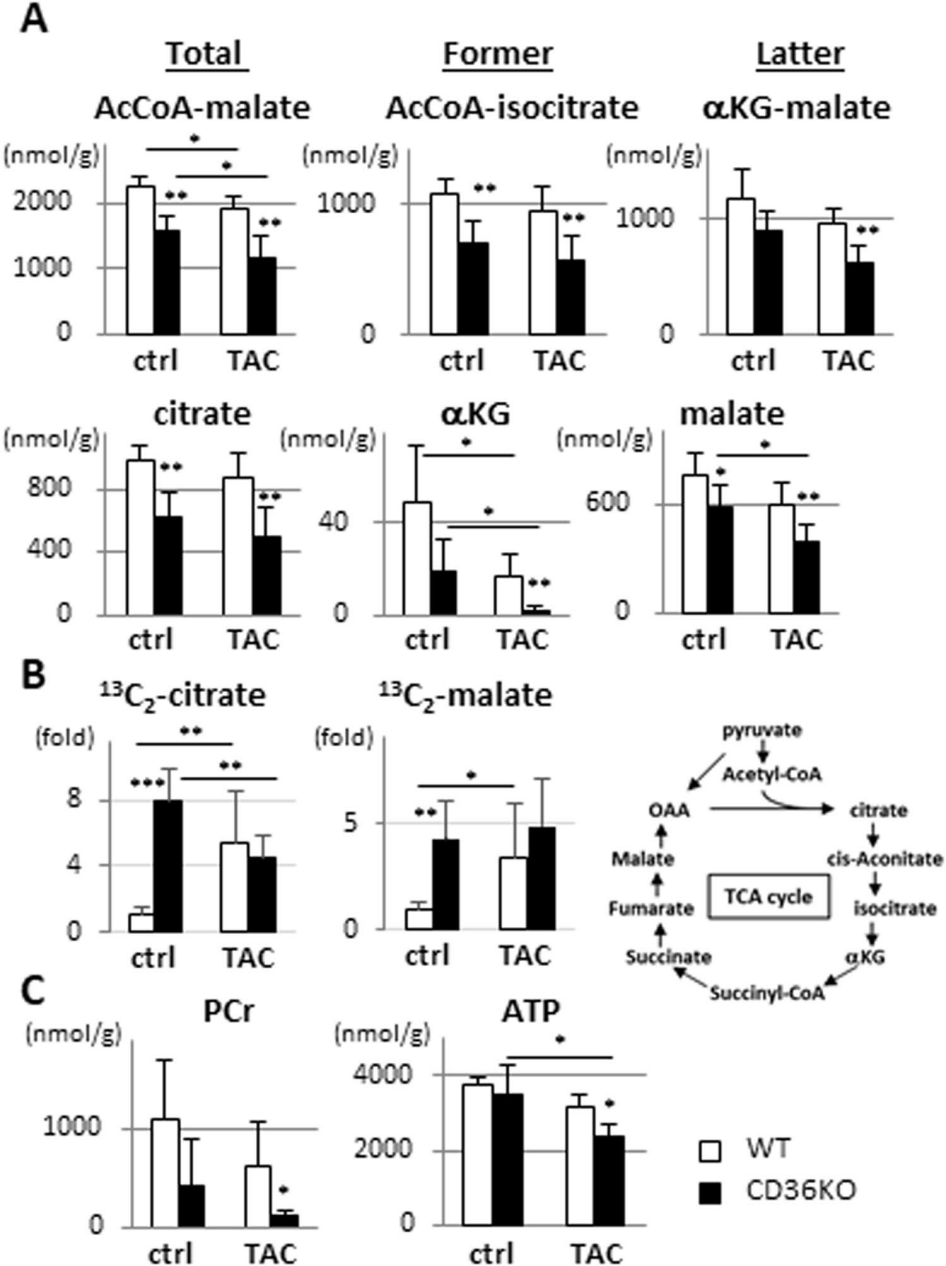
The pool size in the TCA cycle and high energy phosphate were significantly reduced in CD36KO-TAC hearts. Hearts were isolated after a 12 h fast for metabolome analysis. (**A**) Metabolic profiling in the TCA cycle. Note that oxaloacetate (OAA) cannot be detected by the method. TCA cycle, tricarboxylic acid cycle; αKG, α-ketoglutarate (n = 5–7). (**B**) Tracer study with ^13^C_6_-glucose. After a 12 h fast, hearts were isolated 10 min after intraperitoneal injection of ^13^C_6_-glucose (n = 5–7). (**C**) Metabolic profiling in creatine phosphate energy shuttle. PCr, phosphocreatine; ATP, adenosine triphosphate (n = 5–7). ^*^p < 0.05, ^**^p < 0.01, ^***^p < 0.001.

To assess metabolic changes in CD36KO hearts, we performed metabolome analysis. Interestingly, despite a dramatic increase in glucose uptake, total metabolites in glycolysis (G1P to pyruvate) were significantly reduced in CD36KO hearts at baseline, which was not altered in CD36KO-TAC (Fig. 5A). Pyruvate levels were lower in CD36KO-TAC hearts compared to WT-TAC (Fig. 5A). Lower levels of lactate at baseline were marginally increased in CD36KO-TAC (Fig. 5A). Thus, a robust increase in glucose uptake in CD36KO hearts (Fig. 3A) was not accompanied by a parallel increase in steady state levels of metabolites in glycolysis, suggesting that glycolytic flux rate and conversion of pyruvate to lactate or acetyl-CoA in the TCA cycle may be accelerated in CD36KO hearts. Consistent with this assumption, tracer study with ^13^C_6_-glucose revealed that ^13^C_3_-lactate (Fig. 5B) and ^13^C_2_-citrate (Fig. 6B) were significantly increased in CD36KO hearts compared to WT at baseline (2.5-fold and 8-fold, respectively). A more remarkable increase in ^13^C_2_-citrate compared to ^13^C_3_-lactate in CD36KO hearts at baseline suggest that glycolytic products are preferentially utilized in the TCA cycle rather than lactate production in CD36KO hearts. Taken together, our data suggest that glycolytic rate is accelerated in CD36KO hearts and a large part of metabolites through glycolysis enters the TCA cycle with some lactate production.

### Reduced pool size of metabolites in the TCA cycle despite a robust increase in glucose flux

We next analyzed the amounts of metabolites in the TCA cycle. Interestingly, the pool size of total metabolites in the TCA cycle (acetyl-CoA to malate) was significantly reduced in CD36KO hearts at baseline and further reduced by TAC (Fig. 6A). Sum of metabolites in the former part of the TCA cycle (acetyl-CoA to isocitrate) and the latter part (α-ketoglutarate to malate) were similarly reduced in CD36KO hearts (Fig. 6A). Consistent with these findings, each metabolite in the TCA cycle such as citrate, α-ketoglutarate (αKG) and malate were lower in CD36KO mice at baseline and further reduced by TAC (Fig. 6A). Contrary to the reduction of steady state levels of the metabolites, tracing analysis using ^13^C_6_-glucose showed that levels of ^13^C_2_-citrate and ^13^C_2_-malate were markedly elevated in CD36KO hearts at baseline (Fig. 6B), which strongly suggests that glycolytic flux into the TCA cycle is robustly accelerated in CD36KO hearts. Although levels of ^13^C_2_-citrate and ^13^C_2_-malate were significantly elevated in WT-TAC hearts compared to WT at baseline, they were not enhanced in CD36KO-TAC hearts compared to CD36KO at baseline (Fig. 6B). Together, CD36KO hearts displayed an accelerated glycolytic flux into the TCA cycle, but steady-state levels of metabolic intermediates in the TCA cycle were significantly reduced compared with WT mice hearts both before and after TAC. Moreover, accelerated glycolytic flux into the TCA cycle was not further increased after TAC in CD36KO hearts, accounting for severe reduction in the pool size in the TCA cycle after TAC.

### Reduced creatine phosphate energy shuttle in CD36KO-TAC hearts

Pathological cardiac hypertrophy is associated with depletion of energy reserves manifested as a reduction in reserve energy, phosphocreatine (PCr)^3,14^. Further reduction of PCr is accompanied by a decrease in ATP levels in the heart, which is associated with the progression of hypertrophy to heart failure. Consistent with the reduction in the pool size of the TCA cycle (Fig. 6A), PCr levels tended to be reduced in CD36KO mice at baseline, which was significantly suppressed by TAC (Fig. 6C). Although ATP levels were not different between WT and CD36KO mice at baseline, TAC significantly reduced ATP levels in CD36KO hearts, but not in WT hearts (Fig. 6C). These findings suggest that myocardial capability of ATP synthesis is balanced in CD36KO hearts at baseline, but fails to increase under pressure overloaded conditions.

### Improvement of cardiac contractile dysfunction by an MCFA-rich diet in CD36KO-TAC hearts is attributed to efficient replenishment of energy substrate

**Figure 7:**
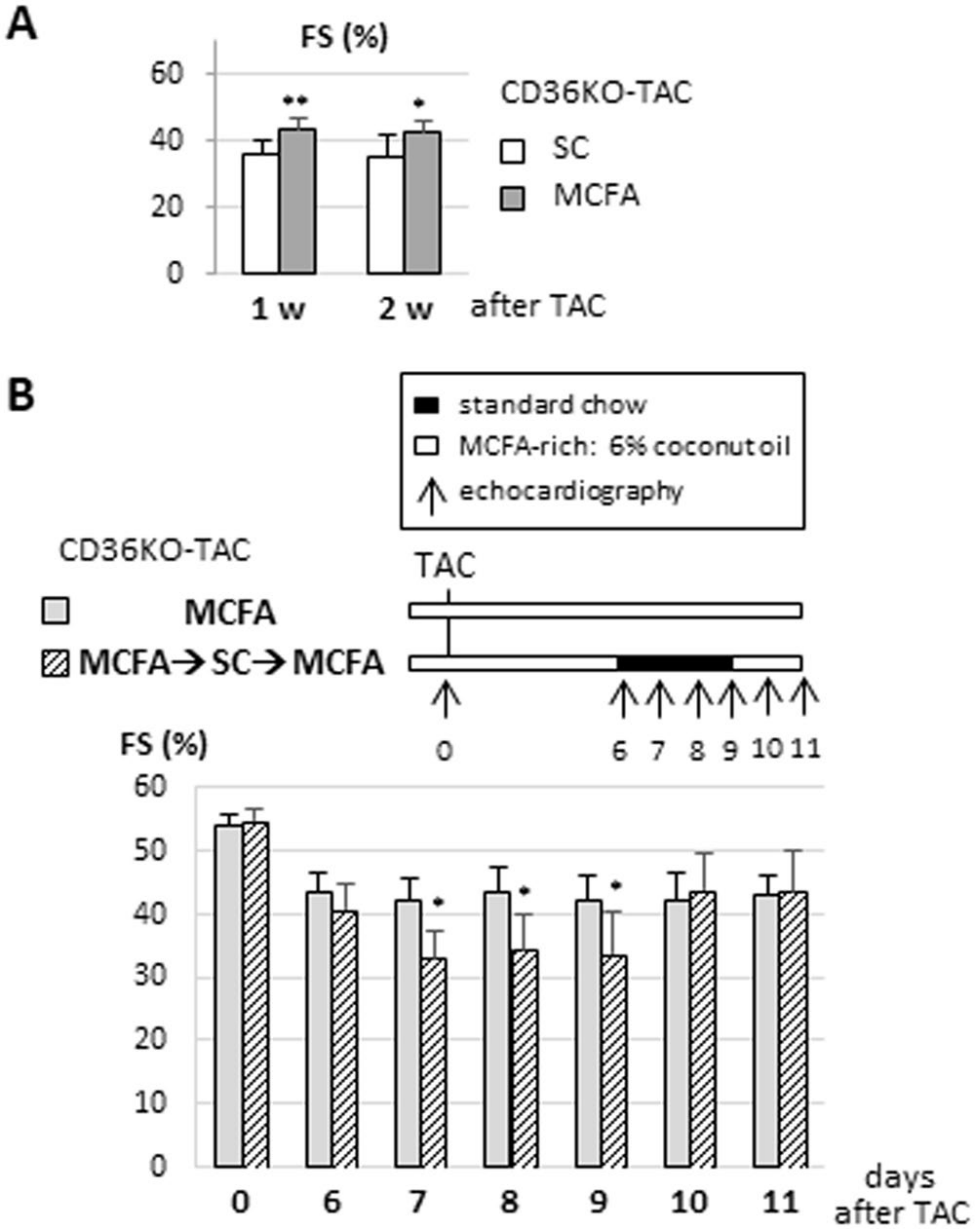
Restoration of cardiac function by an MCFA-rich diet in CD36KO-TAC mice. (**A**) CD36KO-TAC mice were divided into two groups that were fed a standard chow (SC) or an medium-chain FA (MCFA)-rich diet. Cardiac function was evaluated by echocardiography 1 and 2 week after TAC. (**B**) CD36KO-TAC mice were divided into two groups and fed according to the depicted protocol. Cardiac function was evaluated by echocardiography at the indicated time points. ^*^p < 0.05 and ^**^p < 0.01.

Our western blot analysis revealed that expression of mitochondrial proteins such as respiratory chain complex and TFAM (transcription factor A, mitochondrial) was comparable between WT-TAC and CD36KO-TAC hearts (Fig. S2), suggesting that severe cardiac contractile dysfunction in CD36KO-TAC hearts is not due to accelerated mitochondrial dysfunction at least one week after TAC. These findings along with a reduction in the pool size in the TCA cycle prompted us to hypothesize that total fuel supply is insufficient to maintain cardiac contractile function in CD36KO-TAC hearts. MCFA-containing TG have been proposed as metabolic therapy for treating patients with cardiomyopathy suffering from inherited long-chain FA β-oxidation disorder^15^. Importantly, it has been reported that MCFA bypasses CD36 for entry into the cardiomyocytes and that lauric acid (C12:0), the most rich MCFA in coconut oil, is absorbed through intestinal lymphatic vessel, not portal vein^16,17^. Therefore, we next examined whether feeding the mice an MCFA-rich diet (containing 6% coconut oil) can rescue cardiac contractile dysfunction in CD36KO-TAC hearts. As expected, feeding the mice an MCFA-rich diet significantly improved cardiac contraction in CD36KO-TAC hearts compared to those fed a standard chow (SC) diet (Fig. 7A). Cardiac function was comparable between a SC and an MCFA-rich diet groups in WT-TAC hearts (Fig. S3A). Interestingly, improved cardiac contractile function worsened when an MCFA-rich diet was replaced by a SC diet, and cardiac function again improved by refeeding an MCFA-rich diet (Fig. 7B). Serum levels of biochemical parameters including glucose, TG, NEFA and BOH were not elevated in CD36KO-TAC mice fed an MCFA-rich diet compared to those fed a SC diet (Fig. S3B). Interestingly, uptake of glucose and FA, estimated by ^18^F-FDG and ^125^I-BMIPP, respectively, was not affected by an MCFA-rich diet in both WT and CD36KO mice, suggesting that an MCFA-diet improve cardiac function without affecting glucose and FA utilization (Fig. S4). Unexpectedly, however, feeding the CD36KO-TAC mice an MCFA-rich diet did not increase levels of the pool size in the TCA cycle and high energy phosphate despite restoration of cardiac function (Fig. S5). This finding may imply that additional energy supplied by an MCFA-rich diet is utilized for energy production without affecting an increase in the pool size of the TCA cycle (see discussion in detail). Thus, metabolic intervention by feeding the mice an MCFA-rich diet successfully restored cardiac contractile dysfunction in CD36KO-TAC hearts, which suggests that energy insufficiency by reduced uptake of long-chain FA is a primary cause of cardiac contractile dysfunction in our model and that efficient supplementation of energy substrates can rescue cardiac dysfunction due to energy insufficiency.

### Biosynthesis of amino acids and malate-aspartate shuttle in WT-TAC and DKO-TAC hearts

We showed that CD36KO-TAC hearts displays more remarkable hypertrophic and fibrotic responses, which require a large amount of building blocks for biomass synthesis (Fig. 2). These findings raised intriguing possibility that a part of glucose taken up by CD36KO-TAC hearts may be preferentially utilized for biomass synthesis pathways rather than mitochondrial ATP production. Accordingly, we next studied glucose-derived de novo synthesis of non-essential amino acids.

**Figure 8:**
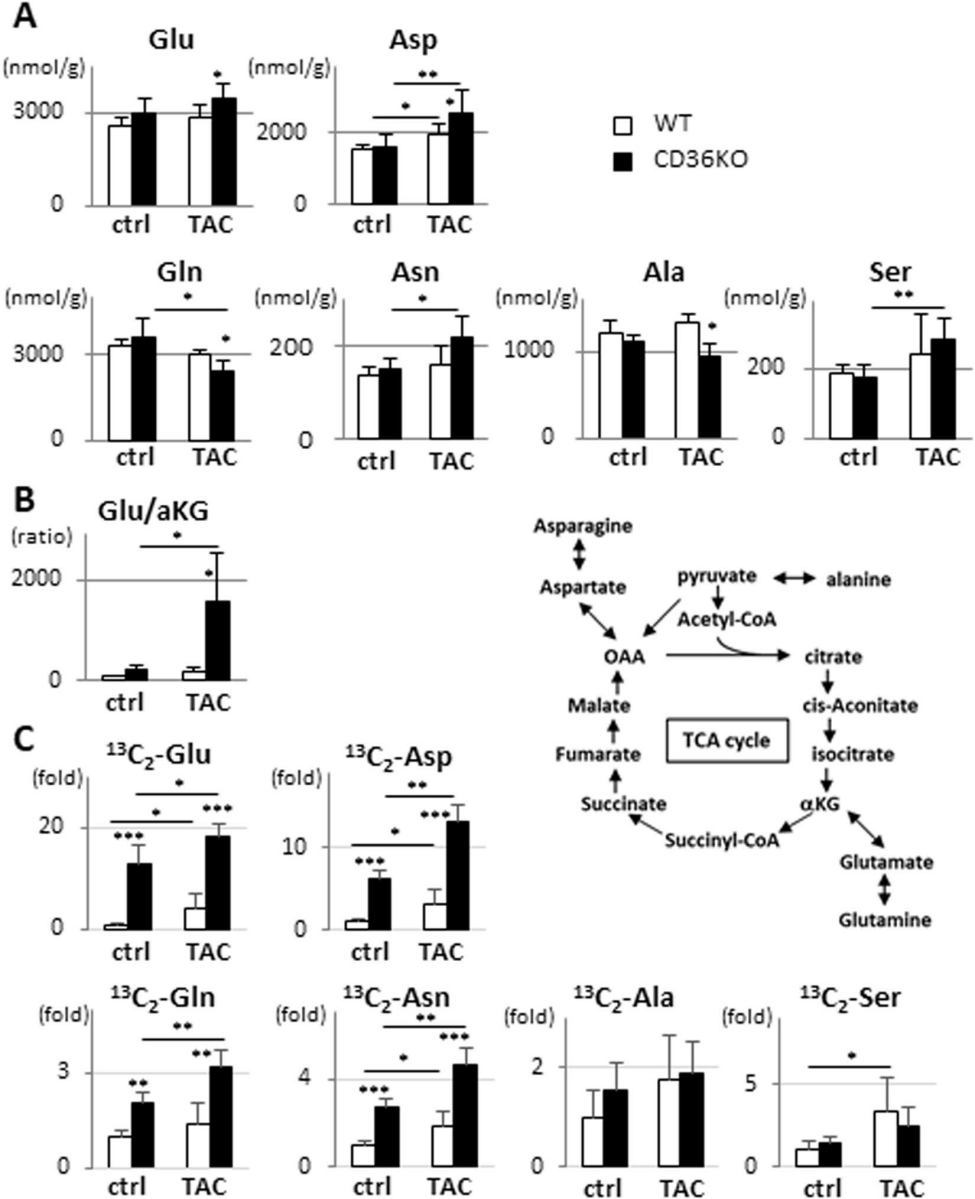
Biosynthesis of non-essential amino acids from glucose seems to be enhanced in CD36KO-TAC hearts. (**A**) Steady state levels of non-essential amino acids in hearts. Hearts were isolated after a 12 h fast for metabolome analysis (n = 5–7). Glutamate (Glu), aspartate (Asp), glutamine (Gln) and asparagine (Asn) can be generated from metabolites in the TCA cycle. Alanine (Ala) and serine (Ser) can be produced from glycolytic intermediates. (**B**) Glu/α-KG, glutamate/α-ketoglutarate ratio (n = 5–7). (**C**) Tracer study with ^13^C_6_-glucose. After a 12 h fast, hearts were isolated 10 min after intraperitoneal injection of ^13^C_6_-glucose (n = 5–7). ^*^p < 0.05, ^**^p < 0.01, ^***^p < 0.001.

Glutamate and aspartate are interconvertible with α-ketoglutarate (αKG) and oxaloacetate (OAA) in the TCA cycle^5,6^, respectively (Fig. 8). Steady state levels of both glutamate and aspartate were significantly elevated in CD36KO-TAC hearts despite a reduction in the pool size in the TCA cycle (Fig. 8A). In particular, glutamate levels were significantly elevated in CD36KO-TAC hearts despite a marked reduction in αKG, a glutamate precursor (Figs 6A and 8A), which was also shown by glutamate/αKG ratio (Fig. 8B). Tracing study with ^13^C_6_-glucose revealed that a large amount of glutamate and aspartate were derived from glucose (Fig. 8C). ^13^C_2_-glutamate and ^13^C_2_-aspartate in CD36KO hearts were 13-fold and 6-fold higher compared with those in WT hearts at baseline, respectively, which were further enhanced by TAC (Fig. 8C). Glutamate and aspartate are components of the malate-aspartate shuttle, which is well-known intercellular compensatory mechanism to maintain cytosolic NAD^+^/NADH redox pair when glycolysis is accelerated^18,19^. Because glycolytic rate, determined by isotopomer analysis (Figs 5B and 6B), is markedly accelerated in CD36KO hearts with and without TAC, the remarkable enrichment of ^13^C_2_-glutamate and ^13^C_2_-aspartate could be tightly coupled to accelerated glycolysis as well as the malate-aspartate shuttle. Steady state levels of other non-essential amino acids such as asparagine and serine were also elevated in CD36KO-TAC hearts although levels of glutamine and alanine were reduced (Fig. 8A). Tracing study with ^13^C_6_-glucose showed that ^13^C_2_-glutamine and ^13^C_2_-asparagine were 2-fold and 3-fold higher compared with those in WT hearts at baseline, respectively, which were further enhanced by TAC (Fig. 8C). Marginal enrichment of ^13^C_2_-alanine and ^13^C_2_-serine was also observed in CD36KO-TAC hearts. These findings demonstrated that besides ATP synthesis, a flux of glucose carbons is directed to the pathways synthesizing amino acids, which is further enhanced by increased workload by TAC. Elevation of steady state levels of several non-essential amino acids with accelerated de novo synthesis from glucose serves as mechanism to link between the biosynthetic requirements of hypertrophic growth of the heart and enhanced diminishment of the pool size in the TCA cycle for ATP production.

## Discussion

Previous study demonstrated that cardiac-specific disruption of the CD36 gene accelerates the progression of pressure overload-induced cardiac hypertrophy to heart failure^13^. CD36KO hearts have been reported to display metabolically compromised phenotype, but precise mechanisms underlying the detrimental effects of CD36KO on cardiac function and metabolisms remains to be determined. Here, we show that CD36-mediated FA uptake is indispensable for sufficient production of high energy phosphate via TCA cycle in the context of pressure overload. Our metabolome analysis collaborated with isotopomer analysis revealed that the pool size of the TCA cycle and high energy phosphate was significantly reduced despite the robust increase in glycolytic flux into the TCA cycle in CD36KO-TAC hearts compared with WT-TAC. Supporting to these data, supplementation of an MCFA-rich diet prevented TAC-induced cardiac dysfunction in CD36KO mice. Furthermore, it is also worth noting that glucose is preferentially utilized for de novo synthesis of non-essential amino acids needed for myocardial hypertrophy in CD36KO-TAC hearts. These findings suggest that long chain FA taken up through CD36 are central energy substrates for sufficient ATP production to maintain cardiac contractile function even under increased workload and that increased glycolytic flux is directed to synthesize biomaterials for structural remodeling rather than ATP production during development of pressure overload-induced hypertrophy.

### CD36KO-TAC hearts are energetically compromised

CD36KO mice have been employed to investigate the role of CD36-mediated FA uptake in the disease conditions such as ischemia/reperfusion injury^20–22^, age-induced cardiomyopathy^23^, adrenergic stress^24^ and pressure overload-induced cardiac hypertrophy^13,25^. The protective effects of CD36 deletion was demonstrated in age-induced cardiomyopathy model^23^ and in cardiac-specific overexpression of PPARα mice^26^. Likewise, CD36 deletion protects the heart from pressure overload-induced heart failure in mice fed western diet^25^ and ishchemia/reperfusion injury^20^. In these mice models, CD36 deletion was effective to decrease excess myocardial lipid oxidation and accumulation. On the other hand, CD36 deletion contributes to cardiac dysfunction in several models such as ischemia/reperfusion, adrenergic stress and pressure overload models^13,22,24^. Importantly, cardiac dysfunction can be improved by supplying MCFA in the models. These studies suggest a causal link between energy deficiency and cardiac dysfunction, which supports our proposal in the current study. Thus, we suggest that reduced FA uptake with compensatory glucose use renders hearts more susceptible to cardiac dysfunction due to energy insufficiency under some diseased conditions. Successful supplementation of MCFA further provides another important notion that ATP synthesis capacity by supplementation of possible fuels is more crucial than the substrate selection for sustaining cardiac energetics and function. Given that CD36 is expressed in cardiomyocytes as well as capillary endothelial cells in the heart^13,20,27,28^, it is also required to distinguish its role of CD36 in cardiomyocytes and capillary endothelial cells in cardiac energetics using cell type-specific CD36KO mice in the future.

### The pool size of the TCA cycle as a marker for energy supply relative to energy expenditure: working hypothesis

The pool size of the TCA cycle was not always consistent with cardiac contractile function in this study. First, cardiac function was preserved in CD36KO mice at rest despite a significant reduction in the pool size compared to WT (Fig. 6A). Second, feeding the CD36KO-TAC mice an MCFA-rich diet did not increase levels of the pool size in the TCA cycle and high energy phosphate despite restoration of cardiac function (Fig. S5). These inconsistent findings could be accounted for by the next hypothesis (Fig. S6).

Energy expenditure (EE) in the heart is minimal at rest and is positively associated with an increase in heart rate, wall stress and contractility^29^. In CD36KO heart, energy supply (ES) is smaller than that in WT heart, which causes reduced pool size in the TCA cycle. However, the reduced ES is sufficient for basal cardiac function because required EE is also small. When EE is elevated by increased workload such as TAC, ES is simultaneously enhanced to meet energy demand in WT heart. However, in CD36KO-TAC heart, limited FA uptake results in diminished ES, leading to reduced EE compared to WT-TAC heart. Because wall stress is elevated by TAC, reduced EE is directly linked to a reduction in contractility. When alternative energy substrate like MCFA is supplemented, ES is enhanced, which increases EE as well as contractility, but not the pool size. Further studies should be warranted to prove this hypothesis.

### The more reduction in FA uptake causes the more severe cardiac dysfunction; comparison between CD36KO and FABP4/5 double KO mice

We have recently reported that mice doubly deficient for fatty acid binding protein 4 (FABP4) and FABP5 (FABP4/5DKO) develop heart failure under pressure overload by TAC^30^. FA uptake in the heart of FABP4/5DKO mice is disturbed at capillary endothelial levels where FABP4/5 are abundantly expressed^31^. In FABP4/5 DKO mice, FA uptake by the heart is reduced by 30–40% with 20-fold increase in glucose uptake at baseline, which is more modest than CD36KO mice; 50% reduction in FA uptake with 35-fold increase in glucose uptake. Interestingly, glucose uptake by the FABP4/5DKO heart is significantly enhanced by TAC, which is two-fold higher than that at baseline. In contrast, enhancement of glucose uptake by TAC in the CD36KO heart was marginal compared to that at baseline, suggesting existence of upper limit of compensatory glucose uptake (Fig. 3A). Cardiac dysfunction seems to be more severe in CD36KO-TAC hearts compared with FABP4/5DKO-TAC^30^ because it was accompanied by a more reduction in survival rate and a more increase in expression of mRNA for cardiac stress markers, *Anp* and *Bnp*. In addition to cardiac contractile dysfunction, compared with FABP4/5DKO mice, we found that CD36 mice displayed more remarkable hypertrophy and fibrosis as evaluated by HW/BW ratio and histochemistry. Given that a robustly increased glycolytic flux is largely utilized for amino acid synthesis and possibly other biomass synthesis such as lipogenesis and nucleotide synthesis, severity of energy deficiency due to reduced FA uptake with compensatory glucose use might also account for the severity of hypertrophy and fibrosis. Collectively, these findings suggest that the more reduction in FA supply causes the more severe cardiac dysfunction with more remarkable structural remodeling under increased workload.

## Conclusions

Taken together, our data indicate that CD36-mediated FA uptake is required for sufficient ATP synthesis to meet the increased energy demand in the context of pressure-overload. A robust increase in glucose uptake fails to serves as efficient compensatory event because glucose is preferentially utilized for biomass synthesis including non-essential amino acids, which aggregates hypertrophic and fibrotic responses. An MCFA-rich diet successfully supplements the fuel and improves cardiac function under pressure overload. Notwithstanding the complexity of human heart failure compared with the mice model, our findings provide the novel insight into the cardiac energy metabolism-function relationship that serves as a potential target for the interventions to modulate “maladaptive” fuel uptake and utilization in heart failure.

## Materials and Methods

All procedures involving animals were approved by the Institutional Animal Care and Use Committee (Gunma University Graduate School of Medicine). All experiments were performed in accordance with the NIH guidelines (Guide for the Care and Use of Laboratory Animals).

### Mice

Mice deficient for *CD36* with the C57BL6j background were generated as described elsewhere^32,33^. Mice were housed in a temperature-controlled room with 12 hours light/12 hours dark cycle and given unrestricted access to water and standard chow (CE-2, Clea Japan). Eight to ten week-old male mice were used.

To study the effects of medium-chain FA (MCFA) on cardiac function, CD36KO-TAC mice were fed a diet enriched in MCFA (AIN-93G, 6% fat from coconut oil, Clea Japan) or standard chow (SC) according to the protocol described in Fig. 7.

### Transverse aortic constriction (TAC), echocardiography and sample collection

Pressure overload was produced by constricting the transverse aorta as previously described^34^. Mice were intubated by modified endotracheal tube and anesthetized by isoflurane (Pfizer, Osaka) using inhalation anesthesia system Narcobit-E (Natsume Co. Ltd., Tokyo) with evaporation flow rate 3–3.5 L/min. *In vivo* cardiac morphology and function was assessed by transthoracic echocardiography (EUB-7500, Hitachi, Tokyo) in conscious mice as described previously^30^. For sample collection, mice were sacrificed 1 week after TAC. To reduce pain and to keep the heart beating, mice were briefly anesthetized in isoflurane-filled box to induce early unconsciousness, and then maintained by mask type isoflurane inhalation system.

### Histology

Myocardium fixed with 10% formalin was analyzed for myocyte hypertrophy and fibrosis as described previously^34^. Tissue was paraffin embedded and sectioned into 10-μm slices. To assess cardiomyocyte cross-sectional area, tissue was stained with wheat germ agglutinin (WGA; plasma membrane staining, Alexa Fluor 488 conjugated). Fibrosis was estimated by Masson’s trichrome stain.

### RNA isolation and real time PCR

Total RNA isolation and quantitative real time-PCR was performed as described previously^31,35^. The expression level of the target gene was normalized to the GAPDH (glyceraldehyde-3-phosphate dehydrogenase) mRNA level. The ready-to-use gene-specific primers for cDNA (perfect real time primer) are purchased from Takara Bio Inc. (Shiga, Japan).

### Biodistribution of ^125^I-BMIPP (15-(p-iodophenyl)-3-(R,S)-methyl pentadecanoic acid) and ^18^F-FDG (2-fluorodeoxyglucose)

The biodistribution of ^125^I-BMIPP and ^18^F-FDG was determined as described previously^31,35^. Mice received intravenous injections of ^125^I-BMIPP (5 kBq) and ^18^F-FDG (100 kBq) via the lateral tail vein in a volume of 100 µl. ^125^I-BMIPP was a gift from Nihon Medi-Physics Co. Ltd., and ^18^F-FDG was obtained from batches that were prepared for clinical PET imaging in Gunma University. The animals were sacrificed at 2 hours after injection. The isolated tissues were weighed and counted in a well-type gamma counter (ARC-7001, ALOKA). Each experiment was performed at least twice.

### Measurement of blood parameters

After a 12 h fast, blood was collected from the retro-orbital plexus to measure serum levels of glucose (Sanwa Kagaku, Aichi, Japan), triacylglycerol (Triglyceride E-test, Wako Chemical, Osaka) and non-esterified fatty acid (NEFA C-test Wako Chemical, Osaka) as described previously^36^.

### Measurement of triglyceride and glycogen in hearts

The ventricles were snap-frozen in liquid nitrogen without delay and pulverized with a mortar and a pestle in liquid nitrogen. The concentration of TG (Triglyceride E-test, Wako Chemical, Osaka) and glycogen (glycogen assay kit, Biovision, CA) in the hearts was determined as described previously^36^.

### Metabolome Analysis by capillary electrophoresis-mass spectrometry (CE-MS)

The mice were anesthetized by isoflurane and the ventricles were immediately removed from the mice. The heart samples were freeze-clamped using aluminum blocks cooled in liquid nitrogen and maintained at −80 °C until use. Metabolome Analyses were carried out as described previously^31,36,37^. A more complete description of the method can be found in the data supplement.

### Tracing study with ^13^C_6_-glucose

After a 12 h fast, ^13^C_6_-glucose (1 mg/g body weight, Sigma Aldrich) was intraperitoneally injected. Ten minutes later, ventricles were isolated and the metabolome analyses were conducted as described previously^30,37^. Mass spectra were collected under selected reaction monitoring mode; m/z 92.30–92.05 for ^13^C_3_-lactate, 193.20–113.10 for ^13^C_2_-citrate, 135.10–116.95 for ^13^C_2_-malate, 149.90–85.10 for ^13^C_2_-glutamate, 149.10–84.15 for ^13^C_2_-glutamine, 136–76.05 for ^13^C_2_-aspartate, 135.10–89.15 for ^13^C_2_-asparagine, and 91.90–44.1 for ^13^C_3_-alanine.

### Statistical analysis

Kaplan-Meyer analysis was used for comparing survival between WT and CD36KO mice after TAC. Comparisons between two groups were performed using an unpaired two tailed Student’s t-test with Welch correction. An unpaired Student’s t-test was performed for each pair of 4 groups and subsequent multiple comparisons were made with use of the Bonferroni method. A p value < 0.05 was considered to be statistically significant. ^*^p < 0.05, ^**^p < 0.01, ^***^p < 0.001.

## Electronic supplementary material

Supplementary Dataset

## Data Availability

The datasets used and/or analyzed during the current study available from the corresponding author on reasonable request.
