# Supplementary material for: Myocardial fatty acid uptake through CD36 is indispensable for sufficient bioenergetic metabolism to prevent progression of pressure overload-induced heart failure
Source: Sci Rep. 2018 Aug 13;8:12035. doi: 10.1038/s41598-018-30616-1 (PMC6089997; doi:10.1038/s41598-018-30616-1)
Supplement: Supplementary file 1 — Supplementary Dataset [file 41598_2018_30616_MOESM1_ESM.pdf]

Supplementary Information

Myocardial fatty acid uptake through CD36 is indispensable for sufficient bioenergetic metabolism to prevent progression of pressure overload-induced heart failure

Yogi Umbarawan, Mas Rizky A.A Syamsunarno, Norimichi Koitabashi, Hideru Obinata, Aiko Yamaguchi, Hirofumi Hanaoka, Takako Hishiki, Noriyo Hayakawa, Motoaki Sano, Hiroaki Sunaga, Hiroki Matsui, Yoshito Tsushima, Makoto Suematsu, Masahiko Kurabayashi, Tatsuya Iso

Supplementary materials and methods

Metabolome Analysis by capillary electrophoresis-mass spectrometry (CE-MS) [1]

The mice were anesthetized by isoflurane and the ventricles were immediately removed from the mice after a 12 h fast. The heart samples were freeze-clamped using aluminum blocks cooled in liquid nitrogen and maintained at -80°C until use. Metabolome Analyses were carried out as described below.

Metabolite Extraction Frozen heart tissue was immediately plunged into methanol (1 ml) that contained internal standards (300 μM each of methionine sulfone for cations and MES for anions) and was homogenized for 1 min to inactivate enzymes. Then, deionized water (500 μl) was added, 300 μl of the solution were transferred to another tube, and 200 μl of chloroform were added, and the mixture was mixed thoroughly. The solution was centrifuged at 12,000 x g for 15 min at 4°C and the 300 μl upper aqueous layer was centrifugally filtered through a Millipore 5-kDa cutoff filter to remove proteins. The filtrate was lyophilized and dissolved in 50 μl of Milli-Q water that contained reference compounds (200 μM each of 3-aminopyrrolidine and trimesate) before CE-MS analysis.

Metabolic Standards All chemical standards were obtained from common commercial sources and were dissolved in Milli-Q (Millipore) water, 0.1 N HCL or 0.1 N NaOH to obtain 10 mM or 100 mM stock solutions. Working standard mixtures were prepared by diluting stock solutions with Milli-Q water just before injection into the CE-MS. The chemicals used were of analytical or reagent grade.

Instruments All CE-MS experiments were performed using an Agilent CE Capillary Electrophoresis System that was equipped with an air pressure pump, an Agilent 1100 series MSD mass spectrometer, and an Agilent 1100 series isocratic high performance liquid chromatography pump, a G1603A Agilent CE-MS adapter kit, and a G1607A Agilent CE-MS sprayer kit (Agilent Technologies). System control, data acquisition, and MSD data evaluation were performed using the G2201AA Agilent Chem Station software for CE-MSD.

CE-MS Conditions for Cationic Metabolites Separations were carried out in a fused silica capillary (50 μm inner diameter x 100 cm total length) that was filled with 1 M formic acid as the electrolyte. Approximately 3 nl of sample solution was injected at 50 mbar for 3 s, and voltage at 30 kV was applied. ESI-MS was conducted in the positive ion mode, and the capillary voltage was set at 4000 V. For MS using the selective ion monitoring mode, deprotonated $[M+H]^+$ ions were monitored for cationic metabolites of interest.

CE-MS Conditions for Anionic Metabolites A cationic polymer-coated SMILE (+) capillary was obtained from Nacalai Tesque (Kyoto, Japan) and was used as the separation capillary (50 μm inner diameter x 100 cm total length). The electrolyte for the CE separation was 50 mM ammonium acetate solution (pH 8.5). Samples were injected with a pressure injection of 50 mbar for 30 s (30 nl). The applied voltage was set at -30 kV. ESI-MS was conducted in the negative ion mode, and the capillary voltage was set at 3500 V. For MS using the selective ion monitoring mode, deprotonated $[M-H]^-$ ions were monitored for anionic metabolites of interest.

Western blot analysis

Western blot analyses were carried out as described elsewhere[2, 3]. Total OXPHOS rodent western blot antibody cocktail (Abcam110413, MA) and antibodies against transcription factor A mitochondial (TFAM, CST, MA), and glyceraldehyde-3-phosphate dehydrogenase (GAPDH, CST 14C10, MA) were used.

Supplementary Figure legends

Figure S1. Representative WGA staining for CSA measurement. Scale bar = 100 μm. WGA, wheat germ agglutinin; CSA cross-sectional area.

Figure S2. Expression levels of mitochondrial protein were comparable between WT-TAC and CD36KO-TAC hearts. Rat mitochondrial protein is provided by the antibody cocktail for respiratory chain complex subunits as control. Lower panels are original pictures without trimming. CV-ATP5A, complex V-ATP synthase subunit alpha; CIII-UQCRC2, complex III-cytochrome b-c1 complex subunit 2; CIV-MTCO1, complex IV-cytochrome c oxidase subunit 1; CII-SDHB, complex II-succinate dehydrogenase subunit B; CI-NDUFB8 complex I-NADH dehydrogenase (ubiquinpone) 1 beta subcomplex subunit 8; TFAM, transcription factor A mitochondrial; GAPDH, glyceraldehyde-3-phosphate dehydrogenase.

Figure S3. A, Cardiac function was estimated by echocardiography 1 and 2 weeks after TAC in WT mice fed a SC or an MCFA-rich diet. Cardiac contractile function was not affected by different diet (n=6-7). FS, fractional shortening. **B**, Serum levels of biochemical parameters

(n=6-7). WT-TAC and CD36KO-TAC mice were fed a SC or an MCFA-rich diet for 2 weeks after TAC. Blood was collected from the retro-orbital plexus with a 4 h fast. TG, triacylglycerol; NEFA, non-esterified fatty acid; BOH, b-hydorxybutyrate. *p<0.05, **p<0.01, ***p<0.001.

Figure S4. WT and CD36KO mice were fed a SC or an MCFA-rich diet for 2 weeks. Uptake of glucose (A) and FA (B) by indicated tissues were estimated by glucose tracer, ^{18}F-FDG, and FA tracer, ^{125}I-BMIPP as described in supplementary materials and methods (n=6). Bld, blood; HRT, heart; Liv, liver; WAT, white adipose tissue; WSM, white skeletal muscle (quadricps); RSM, red skeletal muscle (soleus). *p<0.05, **p<0.01, ***p<0.001.

Figure S5. The pool size of the TCA cycle and creatine phosphate energy shuttle in CD36KO-TAC hearts after feeding a SC diet or an MCFA-rich diet for a week. Hearts were isolated after a 6 h fast for metabolome analysis. PCr, phosphocreatine; ATP, adenosine triphosphate (n=6).

Figure S6. Putative bar graph regarding the pool size of the TCA cycle associated with difference between energy supply (ES) and energy expenditure (EE). There are three points to account for this working hypothesis. First, the pool size of the TCA cycle is likely to be associated with difference between ES and EE. Second, both ES and EE are dynamically regulated in response to workload. Third, EE is positively associated with an increase in heart rate, wall stress and contractility (see discussion in detail).

References

1. Iso T, Maeda K, Hanaoka H, Suga T, Goto K, Syamsunarno MR, Hishiki T, Nagahata Y, Matsui

H, Arai M, Yamaguchi A, Abumrad NA, Sano M, Suematsu M, Endo K, Hotamisligil GS, Kurabayashi M. Capillary endothelial fatty acid binding proteins 4 and 5 play a critical role in fatty acid uptake in heart and skeletal muscle. *Arterioscler Thromb Vasc Biol*. 2013;33:2549-2557

2. Syamsunarno MR, Iso T, Hanaoka H, Yamaguchi A, Obokata M, Koitabashi N, Goto K, Hishiki T, Nagahata Y, Matsui H, Sano M, Kobayashi M, Kikuchi O, Sasaki T, Maeda K, Murakami M, Kitamura T, Suematsu M, Tsushima Y, Endo K, Hotamisligil GS, Kurabayashi M. A critical role of fatty acid binding protein 4 and 5 (fabp4/5) in the systemic response to fasting. *PLoS One*. 2013;8:e79386

3. Goto K, Iso T, Hanaoka H, Yamaguchi A, Suga T, Hattori A, Irie Y, Shinagawa Y, Matsui H, Syamsunarno MR, Matsui M, Haque A, Arai M, Kunimoto F, Yokoyama T, Endo K, Gonzalez FJ, Kurabayashi M. Peroxisome proliferator-activated receptor-gamma in capillary endothelia promotes fatty acid uptake by heart during long-term fasting. *Journal of the American Heart Association*. 2013;2:e004861

Supplementary Figure 1

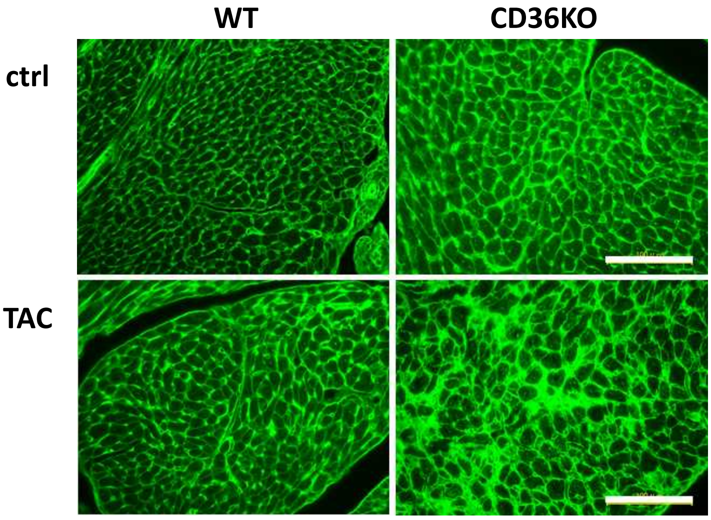

Supplementary Figure 2

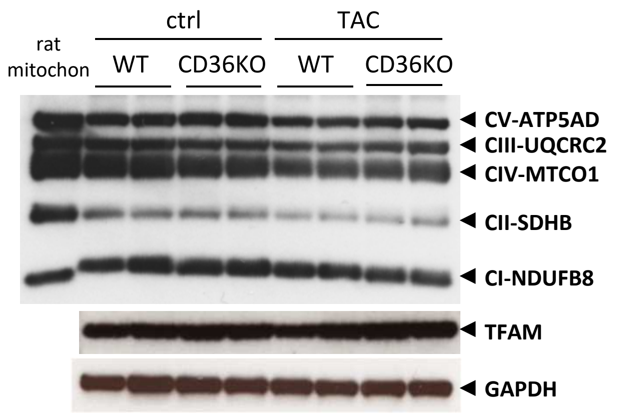

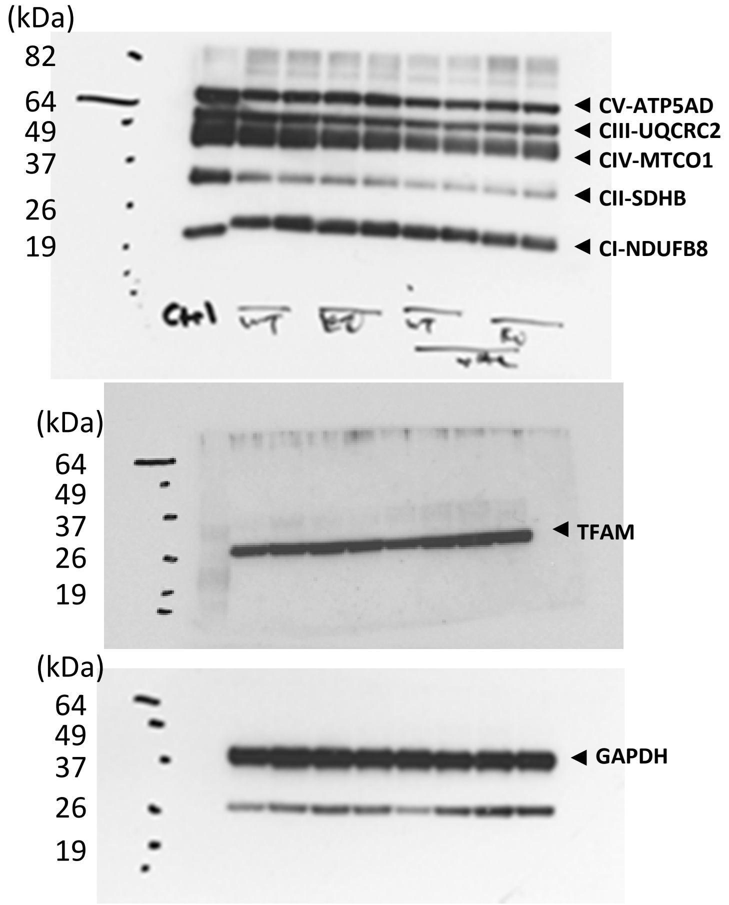

Supplementary Figure 3

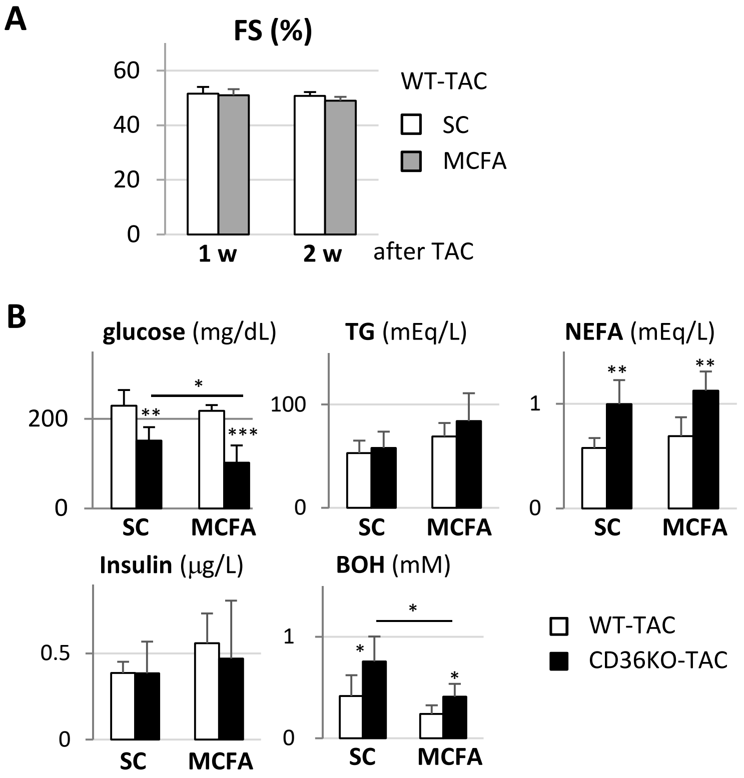

Supplementary Figure 4

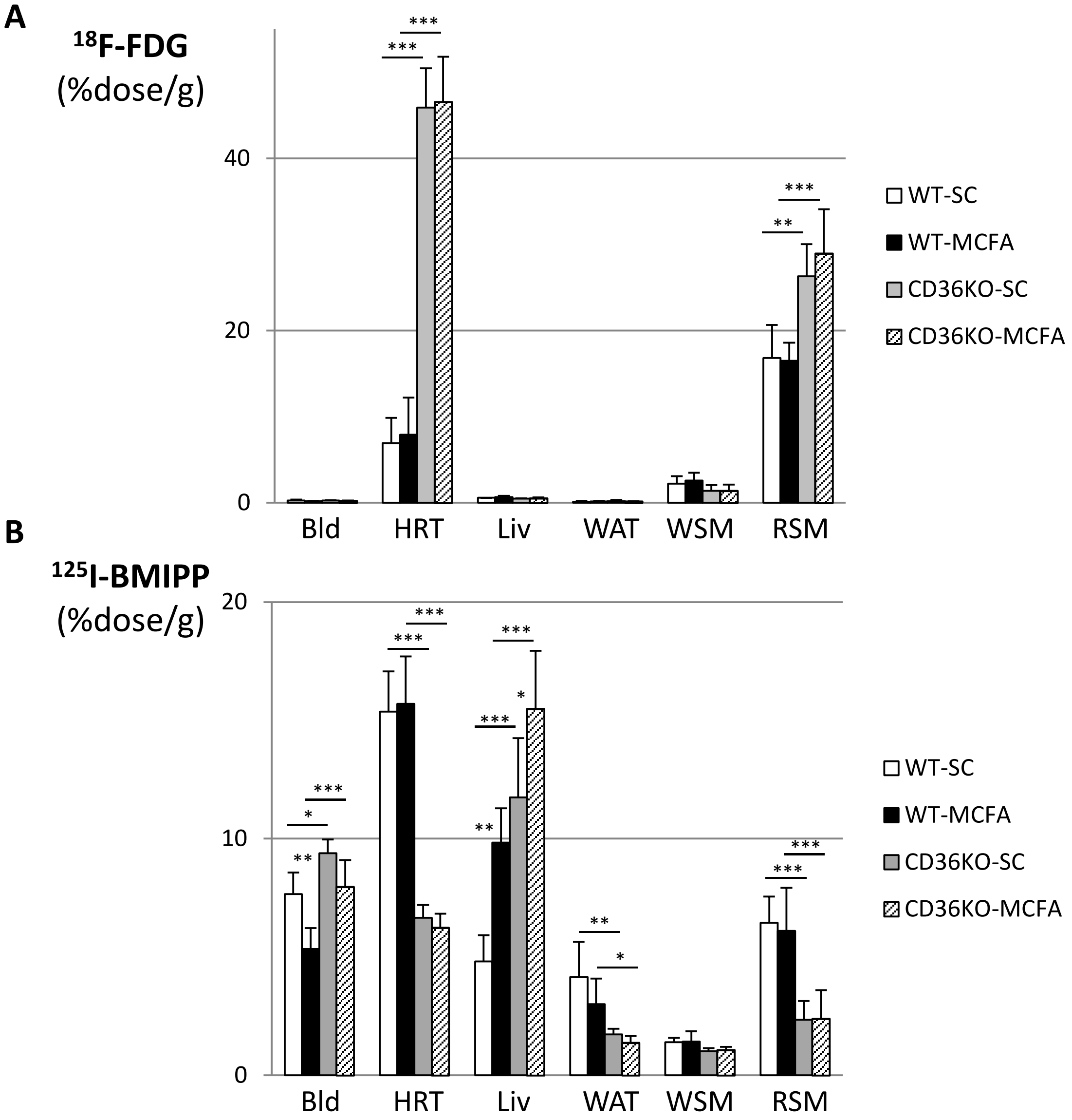

Supplementary Figure 5

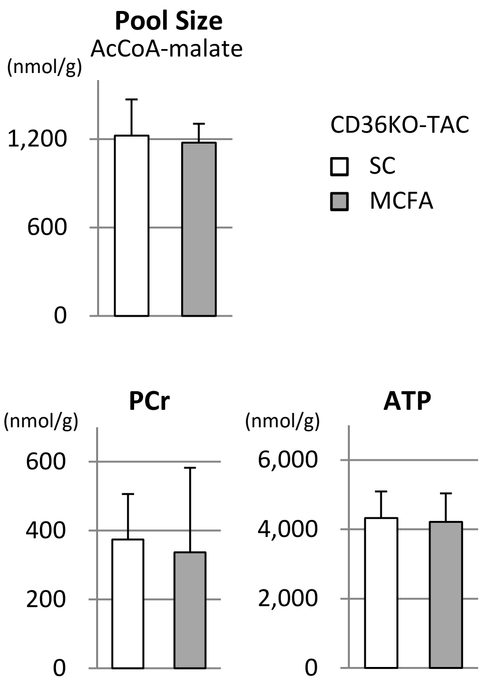

Supplementary Figure 6

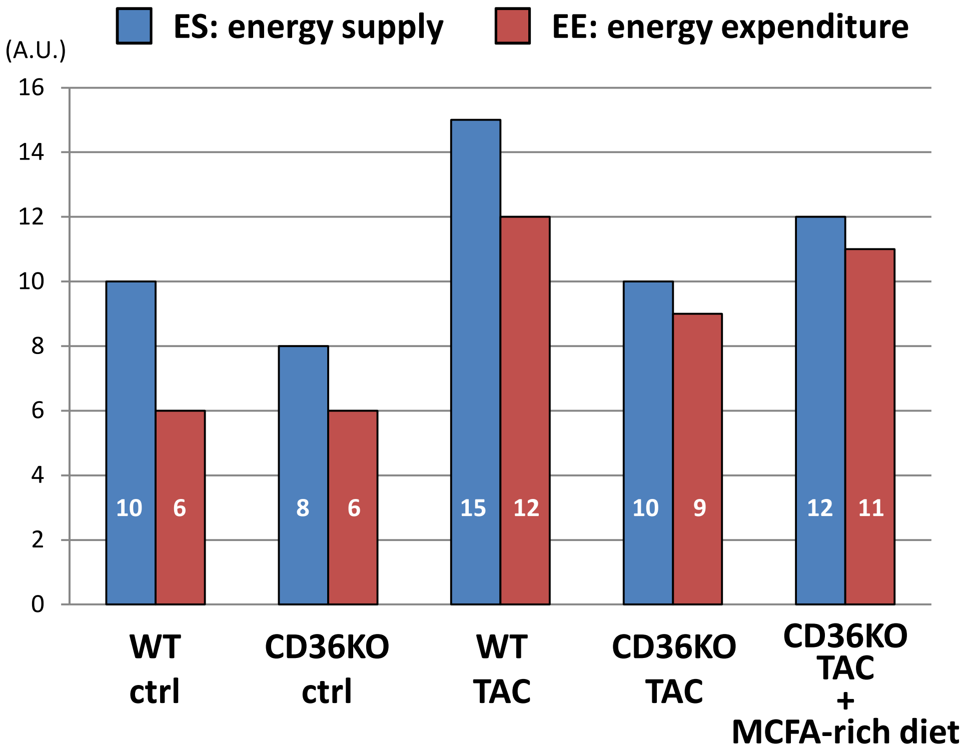

	WT ctrl	CD36KO ctrl	WT TAC	CD36KO TAC	CD36KO TAC + MCFA-rich diet
**Pool Size**	4	2	3	1	1
**Wall Stress**	+	+	++	++	++
**Contractility**	→	→	→	↓	↘
**Heart Rate**	→	→	→	→	→

Pool Size (and high energy phosphate) $\propto$ ES - EE

EE $\propto$ heart rate, wall stress, contractility

Table S1. Comparison of cardiac function between WT and CD36KO mice

	control		TAC					
	Week 0		Week 1		Week 4		Week 8	
	WT	CD36KO	WT	CD36KO	WT	CD36KO	WT	CD36KO
FS (%)	54.5±2.5	55.8±2.5	56.3±2	41.1±4.9***	44.8±2	32.5±6.2***	40.4±5.6	29.6±8.5**
LVDd (mm)	2.9±0.3	2.8±0.2	2.9±0.2	3.4±0.3**	3.2±0.3	3.5±0.8	3.4±0.4	3.5±0.8
LVDs (mm)	1.3±0.2	1.3±0.1	1.3±0.3	2.0±0.3***	1.8±0.2	2.4±0.5**	2.0±0.4	2.7±0.9*
IVSd (mm)	0.9±0.1	0.8±0.1	0.9±0.1	0.9±0.2	1.1±0.1	1.2±0.2	1.1±0.2	1.2±0.2
PWd (mm)	1.1±0.2	1.1±0.2	1.1±0.2	1.0±0.3	1.2±0.1	1.2±0.2	1.2±0.2	1.3±0.3
Heart Rate (bpm)	681±55	679±46	686±42	675±37	696±35	709±30	707±34	712±19

FS, fractional shortening; LVDd, diastolic diameter of left ventricle; LVDs, systolic diameter of left ventricle; IVSd, thickness of interventricular septum in diastole; PWd, thickness of LV posterior wall in diastole; HR, heart rate; bpm, beats per minute. *p<0.05 and **p<0.01 for WT vs CD36KO at the same time point.
